# Health professionals’ perspectives on the impact of COVID-19 on sexual and gender-based violence (SGBV) and SGBV services in Rohingya refugee communities in Bangladesh

**DOI:** 10.1186/s12913-022-08122-y

**Published:** 2022-06-04

**Authors:** Shahanoor Akter Chowdhury, Thomas McHale, Lindsey Green, Ranit Mishori, Chloe Pan, Isabel Fredricks

**Affiliations:** 1 Physicians for Human Rights, PC Culture Housing Society, House #44, Road #11, Block-Kha, Adabor, Mohammadpur, 1207 Dhaka, Bangladesh; 2 Physicians for Human Rights, 434 Massachusetts Ave. Suite 503, Boston, MB 02118 USA; 3 Physicians for Human Rights, Georgetown University School of Medicine, 3900 Reservoir Road, NW, DC 20007 Washington, USA

**Keywords:** COVID-19, Sexual and gender-based violence, Health systems, Sexual violence services, Refugee health, Humanitarian settings, Conflict-affected populations

## Abstract

**Background:**

The COVID-19 pandemic has acutely affected Rohingya refugees living in camps in Cox’s Bazar, Bangladesh. Reported increases in sexual and gender-based violence (SGBV) were attributed in part to pandemic-related public health measures. In addition, the Government of Bangladesh’s restrictions to prevent the spread of COVID-19 have impacted the provision of comprehensive care for survivors of sexual violence. This study sought to understand how the COVID-19 pandemic affected SGBV and the provision of services for Rohingya survivors in Bangladesh.

**Methods:**

Interviews were conducted with 13 professionals who provided or managed health care or related services for Rohingya refugees after the onset of the COVID-19 pandemic in March 2020.

**Results:**

At the outset of the COVID-19 pandemic, organizations observed an increase in the incidences of SGBV. However, health care workers noted that the overall number of survivors formally reporting or accessing services decreased. The pandemic produced multiple challenges that affected health workers’ ability to provide essential care and services to Rohingya survivors, including access to the camps, initial designation of SGBV-related services as non-essential, communications and telehealth, difficulty maintaining confidentiality, and donor pressure. Some emerging best practices were also reported, including engaging Rohingya volunteers to continue services and adapting programming modalities and content to the COVID-19 context.

**Conclusions:**

Comprehensive SGBV services being deemed non-essential by the Government of Bangladesh was a key barrier to providing services to Rohingya survivors. Government restrictions adversely affected the ability of service providers to ensure that comprehensive SGBV care and services were available and accessible. The Government of Bangladesh has not been alone in struggling to balance the needs of displaced populations with the necessary precautions to prevent the spread of COVID-19 and its response can provide lessons to others overseeing the provision of services during epidemics and pandemics in other humanitarian settings. The designation of comprehensive services for survivors of SGBV as essential is vital and should be done early in establishing disease prevention and mitigation strategies.

**Supplementary Information:**

The online version contains supplementary material available at 10.1186/s12913-022-08122-y.

## Background

The COVID-19 pandemic has disrupted millions of lives globally and has acutely affected people in humanitarian crises around the world. Due to restrictions on movement, those living in congregant settings, such as refugee camps, have experienced limitations in their ability to access necessary aid supplies and services, practice social distancing, and access COVID-19 prevention measures. These challenges have been felt acutely by the approximately 855,000 Rohingya refugees living in camps in Cox’s Bazar, Bangladesh at the start of the pandemic in March 2020 [1].

Many countries reported increases in SGBV and intimate partner violence (IPV) during the pandemic and there is a history of past public health emergencies leading to an increased risk of SGBV [2–4]. From the onset of the COVID-19 pandemic, there was global concern about how the pandemic and the necessary public health mitigation measures, including lockdowns and social distancing, would impact the incidence of sexual and gender-based violence (SGBV) [5].

The first confirmed case of SARS-CoV-2 infection in Bangladesh was detected on March 8, 2020 [6]. In late March 2020, the Government of Bangladesh implemented stay-at-home measures for the entire country, including a lockdown for the district of Cox’s Bazar, limiting movement between and out of the nearby camps for refugees and service providers in a bid to halt the spread of the coronavirus [7]. On March 23, 2020 the Office of the Refugee Relief and Repatriation Commissioner (RRRC) issued a directive that all non-essential services in the camps be suspended and that organizations providing essential services, including health care, food distribution, and water and sanitation activities, limit operations to provision of critical services. They were also instructed to try to reduce staff and vehicle movement in and out of the camps [8]. The COVID-19 Response Plan, developed to guide the work of all groups engaged in the Rohingya humanitarian response, was released in July 2020 [9]. The response plan stated that essential health services should include a functioning referral system that links community health with primary health care centers and hospitals; that emergency services must be available and accessible; and that sexual and reproductive health (SRH) services and mental health services must be continued and available to men, women, boys, and girls [9].

In Cox’s Bazar, the lockdowns meant that approximately 80% of humanitarian aid providers were denied entry into refugee camps [10, 11]. Not all aspects of comprehensive SGBV services were deemed essential in the lockdown period [8]. Restrictions on physical movement thus posed challenges to providing and accessing health care services, livelihood opportunities, and support systems for SGBV survivors that are standard in humanitarian crises [12].

Since the outset of the COVID-19 pandemic, organizations working on SGBV prevention and response in the camps have observed an increase in the incidence of SGBV, attributed to heightened stress, close confinement, pandemic-related stressors, and overall trauma [13]. This has been consistent with findings that containment measures increased incidents of SGBV and domestic violence in refugee camps and Bangladesh overall, as well as increasing concerns around personal safety [14–16].

In the camps SGBV services employ a multisectoral approach that provides survivors with various services, often administered through safe spaces for women and girls and/or multipurpose centers. In these spaces, a case worker helps survivors access health care, psychosocial support, protection services, and legal services [17]. Some elements of the multisectoral approach, like health services, individual mental health counseling, and some protection services, were still considered essential during the lockdown period, while others – such as legal services, skill building, and safe spaces – were not considered essential. These services were either not available, available less frequently, only available for individual counseling, or only available for COVID-19 awareness activities [8].

Within the context of the COVID-19 response in the refugee camps in Cox’s Bazar, this study sought to understand how the pandemic affected SGBV among Rohingya refugees, document the impact of COVID-19 on the provision of services for survivors of SGBV and other forms of violence, and highlight emerging best practices identified in the response.

## Methods

Researchers from Physicians for Human Rights (PHR) conducted one-on-one semi-structured interviews with 13 health care workers using a grounded theory approach to understand the experiences of those who provided direct services to Rohingya refugees in Bangladesh. This research was conducted as part of a larger study of sexual violence committed against the Rohingya from the perspectives of health care workers with a specific module related to COVID-19 added during the course of the research [18]. The research team was made up of American and Bangladeshi social scientists and physicians who have experience in humanitarian settings, public health, and responding to SGBV.

Data were collected between April and October 2020. The study received institutional review board approval through Georgetown University (STUDY00001282 and MOD00004144) and an exemption from the PHR Ethics Review Board.

### Study population

The definition of health care providers included community health care workers, paramedics, mental health and psychosocial support (MHPSS) workers, case managers, health project managers, and government health care response managers. Health care providers were included in the sample if they had worked with Rohingya patients in Bangladesh for any period after March 2020.

Respondents were identified through a chain referral sampling approach and contacted by the team via email and telephone. The sampling approach was intended to reach respondents who had provided direct health care or health care services to Rohingya refugees, with priority given to those who worked directly in SGBV care and related services, though not all respondents had specific training or expertise in SGBV response. Data were collected until “saturation “ to maximize variability and ensure sufficient data to identify themes and patterns [19].

### Data instruments

Data were collected using a semi-structured interview guide (Supplementary Material 1) and a brief demographic form. The interview guide covered professional background and contextual details of work with the Rohingya, experiences treating Rohingya patients, experiences specific to physical violence, SGBV, and impacts of the COVID-19 pandemic on patient care and service provision. The demographic form collected information about the respondent including age, gender, nationality, educational background, specialty, and employment information specific to time spent working with Rohingya refugees and current employment.

### Data collection, management, and analysis

Interviews were conducted in Bangla by a female social scientist in Bangladesh and took approximately 60 min. All interviews except one were conducted remotely using Zoom, Skype, and WhatsApp. Each of the remote interviews was conducted in a private space to maintain confidentiality. No incentive was provided to the respondents during and after the data collection process.

Interviews were audio recorded and transcribed into Bangla and then translated into English by qualified transcribers and translators. The interviewer, who is fluent in Bangla and English, reviewed translated transcripts for translation accuracy. Interviews were analyzed using a qualitative data management program (Dedoose) [20]. Four team members reviewed transcribed data and developed a coding dictionary.

The research team reviewed the deidentified coded data to identify themes and patterns responsive to the project’s research objectives and reflective of the data. Data analysis summaries were interpreted within the context of other data and published sources on the impact of COVID-19 on SGBV and services for displaced populations.

## Results

Thirteen professionals who provided health care to Rohingya refugees or managed health-related services after the onset of the COVID-19 pandemic were interviewed. Table 1 describes the cohort.

**Table 1:** Demographic information for respondents

Respondents	
* n* =	13
Respondent gender	
* f* =	9
* m* =	4
Respondent age	
* 25–34 years old*	8
* 35–44 years old*	4
* 45–54 years old*	1
* Over 55 years old*	0
Respondent nationality	
* Bangladesh*	13
Respondent place of employment during period working with Rohingya refugees	
* NGO*	12
* Government*	1
Respondent position while working with Rohingya refugees	
* Health coordinator/project manager/officer*	6
* Sector lead*	2
* Care worker*	1
* Paramedic*	1
* Psychosocial support counselor/case manager*	1
* Assistant Camp in Charge*	1
* Legal associate*	1
* Other*	0
Period in which respondent worked with Rohingya refugees in Bangladesh ^a^	
* 2017*	3
* 2018*	10
* 2019*	13
	13

^a^ Number of respondents known to be working with Rohingya refugees in that calendar year

Health professionals in the sample provided key insights into how COVID-19 impacted overall SGBV/IPV in the camps. They also highlighted challenges faced by health care providers in service delivery related to access and government restrictions, and emerging best practices developed to address these challenges during the COVID-19 response.

### Sexual and gender-based violence and intimate partner violence (IPV) during the COVID-19 pandemic in the camps

Health care professionals anecdotally observed an increase in the incidence of SGBV and felt that measures put in place by the Government of Bangladesh to prevent the spread of COVID-19, specifically movement restrictions which kept individuals in their homes, influenced the increase in SGBV.

*One reason for which this is happening is that they are idly spending time being confined to their home.*

A gender-based violence (GBV) prevention and response officer working with Rohingya survivors since 2018.

The government-imposed lockdown and service restrictions have impacted other factors that respondents linked to IPV. This included economic stressors, mental health triggers, and increased drug use [21]. For example, due to the lockdown and temporary suspension of non-essential services, Rohingya men previously engaged in livelihood programs as day laborers and volunteers have become unemployed.

*The males are more scared. Before, they were actually involved in different types of works, for this very reason, the rate of violence on women has increased. They beat them up for no good reason. Arguments, physical violence, all these types of domestic problems have increased. I believe the situation is pretty much [the] same even in our host community [Bangladeshi communities in Cox’s Bazar].*

A paramedic and psychosocial support worker working in Cox’s Bazar since 2017.

### Service delivery challenges during the response

Despite the suspected increase in the number of cases of SGBV, health care workers noted that the overall number of survivors formally reporting or coming to their centers to access services has decreased. Low utilization of SGBV services in the refugee camps had been noted by many stakeholders in the pre-COVID-19 period. However, the pandemic seems to have led to a further decrease in service utilization.

*A lot less came, a lot less. This number normally is not very much, but in COVID time [at the onset of the pandemic] I will say that in one or two months it was absolutely zero.... So we have a doubt that has this case actually been reduced? I can say one of my assumptions is that this kind of thing didn't actually stop.*

A health coordinator working in the Rohingya response since October 2019.

In addition to a decline in reported incidents, the COVID-19 pandemic produced multiple challenges that impacted health workers’ ability to provide essential care and services to Rohingya survivors of SGBV living within the camps in Bangladesh. These included access issues, challenges related to the initial designation of SGBV-related services as non-essential, communications and telehealth challenges, difficulty maintaining confidentiality, and donor pressure, on top of existing lockdown and social distancing measures [22].

*Our first instruction was that only the essential services would be running in the camp and each service provider would bring their staff members down to 20% in the camp.... That was because we wanted to limit the spread.*

An Assistant Camp in Charge (CiC) working in the Rohingya camps since 2018.

### The impact of public health measures

The Refugee Relief and Repatriation Commissioner (RRRC) instructed service providers to maintain social distancing and health care providers shared how these restrictions necessitated changes in the types of services they provided, the number of people they could see, and how these services were delivered.

*If we are to do it maintaining within three feet distance, it is better not to allow more than five people at a time… There also is a crowd outside and that is also our responsibility to maintain.*

A sexual and reproductive health (SRH) service provider working with Rohingya refugees for more than three years

Outreach and awareness-raising sessions with community members, a key method through which information is shared with Rohingya refugees, were difficult to organize because of the restrictions.

*We cannot hold public meetings…and the main works of our camp are done through public gatherings.*

An Assistant Camp in Charge (CiC) working in the Rohingya camps since 2018.

### Challenges with the designation of SGBV-related services as non-essential

The RRRC’s initial designation of certain services, such as food and health, as essential and the exclusion of SGBV-related care from the list of designated essential services significantly affected health workers’ ability to deliver care. It also impacted their ability to continue ongoing projects for those affected by SGBV, including IPV, in the camps.

*The organizations that follow the policy of the government are … not able to move so far.… Maintaining social distance in any way is not possible in the camp environment.... We've seen that a lot of programs or a lot of projects have stopped half-done.*

An Assistant Camp in Charge (CiC) working in the Rohingya camps since 2018.

A respondent noted indifference on the part of the government regarding the designation of SGBV-related services, including protection services, as non-essential. Some noted that the omission of SGBV-related services from the list of essential services slowed down responses to SGBV.

*The government ... [is] giving more priority to both food and health as life- saving. But, that SGBV is also a part, and that it can also be lifesaving, on that we noticed a little indifference on the part of the government…. If they would have given priority to the GBV part as a lifesaver, we could have made our work faster.*

A GBV service provider working with Rohingya refugees for more than two years.

It was also difficult for service providers to make referrals for sexual and reproductive health (SRH) services.

*It was difficult to provide protection related services.… [If] a person is feeling that s/he has [a] SRHR [sexual and reproductive health and rights] related problem and s/he wants to raise a complaint, now how will s/he raise the complaint? How will s/he do it? If I don't go there and open the community center, s/he can’t come. Or if my staff doesn't go to [do] outreach, s/he can't give information. The information center that we have, the mobile medical service or the information hubs that we have are all closed. That person [the service provider] is not there. The agencies that will refer him/her are not there…. Just some minimal services were there. That way, addressing the SRHR was very challenging.*

A project manager in the protection sector working with Rohingya refugees since 2018.

Though there was not an official lifting of restrictions in the camps from the first lockdown in March 2020, in late 2020 service offerings resumed slowly, with more limited staffing [23]. Over time, respondents noted that, due to advocacy by humanitarian response actors, the full range of protection and SGBV services became designated as essential services in the COVID-19 response plan in the Rohingya camps [9].

### Challenges with health worker access

Restrictions to stop the transmission of COVID-19 made it difficult for service providers to access the camps to provide care and support to survivors of SGBV. When they could access the camps, the duration of time available to deliver service to survivors was also reduced.

*If I talk from [an] institutional perspective, you know that we cannot just send all our cars to the camp even if we want to because we have some restrictions from RRRC.… Where we used to use 100 cars, now we have 40 cars assigned and registered for the whole [organization name redacted]. So those have been allocated for every program according to the number of staff. That's why not all our staff, those that are involved in providing GBV services, can go to the camp.*

A GBV service provider working with Rohingya refugees for more than two years.

The changes in service availability led to confusion and the spread of misinformation about the availability of services during the lockdown period. Misinformation posed an additional challenge to service providers and impacted their ability to reach survivors of SGBV.

*[Rohingya refugees thought] service won’t be provided. Some … got news from somewhere that the service is now closed. This was also one of the confusions, which we all had to work on. We had to convince them that the service is not closed in any way.*

A health coordinator working in the Rohingya response since October 2019.

Where services were still delivered, movement restrictions between camps limited survivors’ ability to access centers

*But still they are actually not getting all the services properly for maintaining social distance in this way and due to their movement restriction. For example, psychosocial support, case management. Because they can't actually come to take our services, they can't reach the centers.*

A gender based violence (GBV) service provider working with Rohingya refugees for over two years.

Furthermore, because non-essential programming, such as skill development, was not running, it was more challenging for survivors to access services discreetly, as they often would covertly access SGBV services while attending skill-building sessions.

### Donor expectations

There was pressure from donors to continue providing services during the pandemic, despite local restrictions. It posed a challenge for service providers, who had to follow RRRC orders and guidelines rather than meeting donor expectations and requirements. A respondent shared a situation where donor imperatives and government restrictions were at odds.

*We are here to provide the service. We also have an accountability question because you know we are to work for donor agencies. Donors put pressure on us [asking] why we can't provide [the services]. Now we have to respect our local administration as they are serving here day and night. It’s them who have to give us a wave separately, as it will be monitored by them as well. But it’s us who will provide the services.*

A project manager in the protection sector working with Rohingya refugees since 2018.

### Challenges with communication and providing telehealth services

Health professionals had difficulty communicating with patients and with one another due to mobile network restrictions, which affected their ability to coordinate and provide counseling, survivor outreach, and other critical services and referrals. Respondents described that Rohingya refugees, including Rohingya staff/volunteers, living in camps in Bangladesh were not legally allowed to own SIM cards due to internet and telecommunications restrictions put in place by the Government of Bangladesh before the COVID-19 pandemic [24]. The government has explained these bans as being for security reasons, but they have been widely critiqued by Rohingya civil society, human rights, and humanitarian groups [25–27].

Like clinicians in the rest of the world, health workers had to pivot to offering services via telehealth once COVID-19 struck. Challenges using this service delivery method in the refugee camps were compounded by internet problems, limited access to cell phones (especially for women), and lack of safe spaces, among others.

*With the survivors, there is one problem that is we cannot use our mobile phones. CIC [Camp in Charge] had issued us an embargo, that we do not contact (the survivors) by using mobile phones, although many organizations do contact using mobile phones, but we actually follow the rules very strictly, so that we do not create any further problem in any other way.*

A GBV prevention and response officer working with Rohingya survivors since 2018.

*In case of phone counselling, sometimes we don’t find [a] proper network connection. So, that was a point. We didn’t get proper network sometimes, so couldn’t talk properly.*

A clinical psychologist working with Rohingya refugees since 2017.

Despite the communications restrictions, many organizations still attempted to deliver what services they could remotely. However, gendered access to communications made access to telehealth services difficult. Respondents shared that women often do not have access to mobile devices and had to use their husband’s mobile phones to access telehealth and phone-based psychosocial support.

*So what we are doing is we are providing them hotline numbers so that they can somehow reach us through mobile…. We will surely call them back and provide psychosocial support or see if there is a case to be taken.... But there also is a challenge and that is [most] mobile owners are male persons of the households. So women’s access to mobile phones is tough.*

A gender-based violence (GBV) service provider working with Rohingya refugees for more than two years.

### Lack of safe spaces

Even if some patients could physically access mobile phones, some patients were still unable to access telehealth services because of lack of safe and confidential spaces to make those calls or because they needed permission from male family members to access the phone.

*Many organizations started to provide mental health services by introducing ‘Telemental Health Service,’ but I will say that this process did not turn out to be very fruitful from the perspective of the camps because, for one, not all of them have access to a mobile phone. On the other hand, … it is not safe for the intimate partner [in cases of IPV], as the intimate partner can see from where he is receiving the call, which could aggravate the situation even further. So, at that time, actually our follow-up number was not that high.*

A GBV prevention and response officer working with Rohingya survivors since 2018.

### Challenges maintaining confidentiality

The closure of safe spaces for women and girls and other locations where survivors could access SGBV- and IPV-related services without being identified made it difficult to maintain confidentiality.

*The [redacted] house is just in front of our community center, so we had to make it look like we are providing a complete service where the girls are coming for attending sessions, putting henna paste on each other’s hands, and talking to each other. In this way, along with all these, if a survivor reaches us out through the hotline, she can easily say that she is coming here for [a] session, but in reality, she would be receiving the case management services.... Our outdoor activities were closed, which imposed a huge challenge because now we could not visit homes directly, as we used to do before…. One of my clients … we know that there is risk in visiting her house, so we cannot approach her, which we used to do before.*

A GBV prevention and response officer working with Rohingya survivors since 2018.

The safe spaces where survivors would be able to anonymously access SGBV-related services while also accessing non-related services were closed. Therefore, many women risked revealing their experiences of SGBV to others when engaging with case workers, volunteers, and other service providers in public or in their homes. Some survivors were lost to follow-up because they could not interact with a health professional at home in the presence of their abusive spouse.

## Emerging best practices

Despite the deep challenges health professionals faced while delivering SGBV-related care and services during the COVID-19 pandemic, some emerging best practices were reported by those interviewed for this study.

### Rohingya volunteers

Many participants in this study noted that Rohingya volunteers who lived in the camps were essential to continuing services during the COVID-19 pandemic. Staff who could not access the camps due to government restrictions relied on Rohingya volunteers to follow up with Rohingya beneficiaries living in the camps. Per pre-pandemic guidance, Rohingya refugees were given cash payments for their work [28]. Community-based Rohingya volunteers were so essential to the continued delivery of services that some organizations increased the number of community volunteers to conduct household visits.

*So, with that in mind, we've actually increased community volunteers, who, rather than providing WGSS [women and girls safe spaces] or center-based services, will visit the households and further refer if they find any incidents from there.*

A GBV service provider working with Rohingya refugees for more than two years.

*In the lockdown situation, we often could not directly work and at those moments we utilized the PSS [psychosocial support] volunteers much more. That means we involved them to bring up the issues…. The referrals were done over telephone…. They are trained up in that manner. The community people rely much on them since they belong to the same community.*

A psychosocial support officer and case manager working in a camp since 2018.

### Adapted programming modalities and content

To be responsive to the evolving context service providers in the Rohingya camps came up with different program modalities and content adapted to the pandemic.

*Due to the COVID [-19] situation, we diverted that skill training a bit and went into mask making so that the vulnerable women and girls can involve with mask making and receive such trainings. So, this mask making is under the livelihood sector, although we are working collaboratively with them. And we are giving them a specific amount for each mask making so that they are financially sufficient. So, basically, in these three aspects we actually work in GBV program. And at the same time, there is referral mechanism. We probably do not have many types of services, especially in terms of special health service or SGBV, for example, the clinical mechanisms or if anyone needs safe shelter. So, regarding these, we provide the referral service strongly.*

A gender based violence (GBV) service provider working with Rohingya refugees for more than two years.

One example of adaptive programming sought to shift gender roles during the pandemic to make men more active in household activities.

*What we did then was that we adapted some materials from WHO, then we developed a module on ‘how to address anxiety during COVID-19’ and we move forward with a model like that.… Since they spend a lot of time at home during this time and many of them have no work to do, [we developed a module] how they could contribute to the household works and could create a better environment at home, by being at home. This model gained a lot of popularity among the males.... They were assigned with a homework and there was a fixed group there. In that fixed group, things were shared, like, I have taken care of my baby throughout the day. My wife did the cooking, and I brought all the required water, I did the shopping (grocery, raw food) today. Like this, slowly, by doing this kind of sharing activities, they themselves became interested in how they could help or contribute to the household works.*

A GBV prevention and response officer working with Rohingya survivors since 2018.

## Discussion

The COVID-19 pandemic has had an enormous impact on Rohingya refugees in general and on SGBV survivors in particular [29–32]. This study shows that comprehensive SGBV services being deemed non-essential by the Government of Bangladesh was one of the key barriers to providing services to Rohingya survivors of SGBV. These government-imposed restrictions adversely affected the ability of service providers to ensure that comprehensive SGBV care and services were available and accessible to survivors, despite these services having been identified early in the pandemic by UNFPA, WHO, and other international bodies as essential [33].

The lockdowns and associated restrictions in 2020 had a clear impact on the provision of services for survivors [34]. The experiences shared by respondents indicate that comprehensive services for survivors were not adequately prioritized in response plans, well-resourced, or made accessible for many survivors in the context of social distancing measures – despite these all being cornerstones of WHO’s recommendations for response [33]. Though social distancing and other precautions were necessary to prevent the spread of COVID-19, the containment measures put in place did not have to stop the continuation of services that comprise a multisectoral response to SGBV [35–37]. Organizations developed creative solutions to continue care and support for survivors. However, the Government of Bangladesh continued to enforce policies that deprioritized and undermined comprehensive services for survivors of SGBV [38, 39]. We see similar challenges reflected in the experiences of other displaced populations including increased risk of COVID-19, limited access to services, and increased difficulty accessing information; however this study is unique in that it seeks to understand the impact of the Government of Bangladesh’s restrictions on services for SGBV survivors [40–42].

The findings of this study are significant, as they reinforce existing findings related to how restrictions put in place by the Government of Bangladesh blocked an effective response to SGBV survivors.[43–45]. Some elements of SGBV services, such as individual counseling and health services to treat survivors’ physical needs, were deemed essential and allowed to continue. However, not all of the elements of a survivor-centered SGBV response were deemed essential – such as access to legal services and to women-friendly spaces, which were closed for all but COVID-related activities. Not designating all services as essential created additional substantial barriers to survivors seeking even the most basic care. Our data also provide a more nuanced understanding of how COVID-19 restrictions, existing restrictions, and long-standing challenges in the Rohingya camps conspired to aggravate the impacts of COVID-19 on the ability of Rohingya SGBV survivors to seek care and services [30]. SGBV survivors living in Rohingya refugee camps in Cox’s Bazar faced challenges accessing services prior to COVID-19 [18]. These data show that challenges for SGBV survivors have only been further compounded by policies and practices in the COVID-19 response that systematically deprioritized the needs to survivors.

As the COVID-19 pandemic has continued, so too have policies that deprioritize the needs of SGBV survivors. In April 2021, following a steep increase in COVID-19 transmission rates, the Government of Bangladesh imposed new containment and risk mitigation measures, including a complete lockdown of the five Rohingya camps with the highest number of cases and heavier containment and risk mitigation measures being imposed on the remaining 29 camps [39]. Under the new guidance, only health activities and food and gas distribution were allowed. All learning centers, training facilities, workshops, and awareness sessions were canceled until further notice. Mental health and psychosocial support services, SGBV-related activities, all child-friendly and women-friendly spaces, and social protection activities were stopped [38, 39]. While COVID-19 measures are regularly modified based on the changing local and national pandemic conditions, and continue to be necessary to reduce excess morbidity and mortality from the disease, these types of recurring restrictions indicate that steep barriers consistently exist for SGBV survivors seeking comprehensive services. As the pandemic has evolved, so too has the understanding of how lockdown restrictions have impacted SGBV survivors’ access to services, therefore we would expect to see adapted and revised lockdown restrictions take these lessons learned from the early stages of the pandemic into account.

Our findings are also aligned with guidance and recommendations from several multinational organizations on addressing SGBV within the COVID-19 response, specifically recommendations to ensure that care is safe, accessible, and acceptable and that services for women who experience violence during COVID-19 should be strengthened [46, 47]. Before the pandemic, survivors could access comprehensive, survivor-centered care and treatment without having to disclose their SGBV history.

While SGBV services were technically made available during the pandemic, and the government provided access and updated referrals for SGBV survivors, our data show that they were not available in ways that were accessible to all survivors without disclosing their history of SGBV and potentially exposing themselves to greater risk. Government restrictions were therefore both not aligned with existing international recommendations and best practices and also not aligned with the key principles of the availability, accessibility, acceptability, and quality (AAAQ) framework, central to the fulfillment of the right to health [48]. Furthermore, the restrictions imposed by the Government of Bangladesh should be viewed as a form of violence in themselves and an extension of the trauma experienced by the Rohingya, as the restrictions have perpetuated known elements of structural violence by increasing uncertainty and exacerbating individual vulnerabilities [49].

### Limitations

The data for this study were collected in 2020 and the pandemic has continued to evolve. Therefore, we must interpret the data as time-limited and specific to the initial response period and not as representative of the ongoing response. There are also limitations to the generalizability of these data to the experiences of health care workers and organizations involved in the COVID-19 response in Cox’s Bazar because our key informant sample was small and not random.

Data interpretation and analysis are subject to interpretation bias introduced by the researchers. To address this challenge, the research team was chosen from people with various cultural and professional backgrounds and worked collaboratively.

The study respondents represented a diversity of geographic and cultural backgrounds but did not include Rohingya respondents. Therefore, observations regarding culturally interpreted behaviors were mediated through multiple cultural and linguistic filters and were highly contextual.

## Conclusions and Recommendations

The Government of Bangladesh has not been alone in struggling to balance the needs of displaced populations with the necessary precautions to prevent the spread of COVID-19 [50]. The government of Bangladesh’s response to the pandemic can provide lessons to other governments and bodies overseeing the provision of services during epidemics and pandemics in other humanitarian settings. The experiences of Rohingya survivors of SGBV and those providing them care in Bangladesh show that the designation of services for survivors of SGBV as essential is important and should be done early in establishing disease prevention and mitigation strategies.

These findings should be used to inform new action plans for the Government of Bangladesh for pandemic preparedness and responses to similar crises. In addition, in the short term, the Government of Bangladesh and the international community must ensure that Rohingya survivors of SGBV living in the camps have access to dignified and high-quality care and services. These lessons should also be applied to frameworks and strategies for provision of services Rohingya refugee populations in Bangladesh, including those who have been relocated to Bhasan Char [51]. Access to such services requires timely and accurate information that empowers people to make decisions about their health. In the current pandemic context, for example, the communication restrictions imposed by the Bangladeshi Government hamper the information access and health sector coordination that are key for effective prevention and response. These restrictions should be lifted and discontinued.

Future SGBV-related pandemic preparedness should include specific plans to provide continuous comprehensive services to survivors of SGBV without unnecessary barriers to access and which acknowledge the particular circumstances of survivors living in congregate settings, such as refugee and internally displaced persons’ camps. This study highlights the important role of Rohingya volunteers and community health workers in ensuring the continuity of services for survivors of SGBV during the COVID-19 pandemic. Future pandemic prevention and mitigation plans should include the participation and a formal role for empowered and engaged community members to ensure the inclusion of services identified as crucial by the community as well as the provision of services in a way that is guided by community needs. As SGBV services are critical and essential during the COVID-19 pandemic, pandemic and outbreak response plans should be guided by existing health and human rights principles, including the availability, accessibility, acceptability, and quality (AAAQ) framework [48, 52]. This should be seen as a key lesson learned from the COVID-19 pandemic and a cornerstone of future rights-based responses to global health crises.

## Supplementary Information

**Additional file 1:** 

## Data Availability

The deidentified data used for this analysis can be made available upon reasonable request, in accordance with the study’s informed consent process. For further information, contact the corresponding author.

## Abbreviations

SGBV: Sexual and gender-based violence
SRH: Sexual and reproductive health
SRHR: Sexual and reproductive health and rights
IPV: Intimate partner violence
RRRC: The Office of the Refugee Relief and Repatriation Commissioner
PHR: Physicians for Human Rights
PSS: Psychosocial support
MHPSS: Mental health and psychosocial support
CiC: Camp in Charge
SRH: Sexual and reproductive health
GBV: Gender-based violence
AAAQ: Availability, accessibility, acceptability and quality
