# Supplementary material for: Health professionals’ perspectives on the impact of COVID-19 on sexual and gender-based violence (SGBV) and SGBV services in Rohingya refugee communities in Bangladesh
Source: BMC Health Serv Res. 2022 Jun 4;22:743. doi: 10.1186/s12913-022-08122-y (PMC9166216; doi:10.1186/s12913-022-08122-y)
Supplement: Supplementary file 1 — Additional file 1: [file 12913_2022_8122_MOESM1_ESM.pdf]

Guide for conducting semi-structured interviews of clinicians who encountered Rohingya refugees in Bangladesh

This section to be completed by the interviewer	
Date	
Primary Interviewer Name	
Location of interview	
Unique Identifying Code	
Audio record number	
Interpreter name (if applicable)	

[NOTE TO INTERVIEWER: Read Verbal Consent and sign/date consent statement before proceeding.]

A. INCLUSION CRITERIA

1. Are you a healthcare worker who treated Rohingya refugees in Bangladesh after August of 2017?
    1. YES → Continue to next section.
    2. NO → End interview.

[INTERVIEWER: I will start by asking you a number of questions about your personal and professional background and place of work as well as experiences related to sexual and gender-based violence within your Rohingya patient population]

B. BACKGROUND

1. Can you please describe your professional background in some more detail?

    POTENTIAL PROBES: Can you describe the setting of your work in Bangladesh (i.e. urban/rural, type of health facility? What was/is the general profile of your Rohingya patient population? (ethnicity, ages, genders, SES, level of education).

2. Can you please describe what you have seen in your patient population in terms of sexual and/or gender-based violence?

    POTENTIAL PROBES: What kinds of sexual and/or gender-based violence have you seen? Other than women, have you heard about other genders who have experienced sexual and/or gender-based violence (e.g., boys/men, transgenders)? Can you describe the experiences of your Rohingya patients who suffered sexual and/or gender-based violence in the camps? What kinds of sexual and/or gender-based violence have you seen that they have faced in the camps?

C. COVID 19

[INTERVIEWER: The following questions will be more about your experience related to the COVID 19 pandemic and its impact on your work]

1. Overall, how has the COVID-19 pandemic impacted your work in terms of providing health services to Rohingya refugees? How do you think COVID 19 has impacted your patients?

2. Has the COVID-19 pandemic impacted sexual and gender-based violence within the Rohingya communities? Has the level of patients reporting sexual and gender-based violence changed since the start of the COVID-19 pandemic? If so how?

3. How have services for sexual and gender-based violence been impacted by COVID-19? How do you think COVID-19 has impacted sexual and gender-based survivors in the Rohingya community?

4. What challenges have you encountered providing health care to Rohingya refugees? Providing care to sexual and gender-based violence survivors?

    POTENTIAL PROBES: Can you describe those challenges? How did you address those challenges? What kind of institutional support would you need to help you provide the best care for individuals from this population?

5. How have the challenges you have faced providing care for Rohingya refugees changed during the COVID pandemic?

6. How has the communication infrastructure (e.g., network connectivity, internet blackouts) impacted health workers care coordination with other health workers? Care coordination between organizations?

7. Overall, how do you feel that the communication infrastructure has affected patients access to health care? Access to care among sexual and gender-based survivors?

8. How has the communication infrastructure impacted patients access to health information?

POTENTIAL PROBES: What methods of communication been used to communicate with camp residents about COVID 19? What information is being provided (e.g., preventive practices, disease symptoms? health resources?) What factors have affected these communication efforts? (challenges and facilitators)

9. Is there anything else you want to say regarding your experiences working with Rohingya refugees during the COVID 19 pandemic?

[INTERVIEWER: Thank you for your time.]
